# Supplementary material for: Predicting individual traits from models of brain dynamics accurately and reliably using the Fisher kernel
Source: eLife. 2025 Jan 31;13:RP95125. doi: 10.7554/eLife.95125 (PMC11785372; doi:10.7554/eLife.95125)
Supplement: Supplementary file 1. [file elife-95125-supp1.docx]

**Ahrends, Woolrich & Vidaurre (2023): Predicting individual traits from models of brain dynamics accurately and reliably using the Fisher kernel – Supplementary File 1**

**Supplementary File 1a** Behavioural variables

| **Variable no.** | **Column Header** | **Full Display name** | **Assessment** | **HCP Var. no.** |
| --- | --- | --- | --- | --- |
| 1 | Age_in_Yrs | Age in Years | Demographic | 4 |
| 2 | MMSE Score | Mini Mental Status Exam Total Score | Cognitive Status (Mini Mental Status Exam) | 196 |
| 3 | PicSeq_Unadj | NIH Toolbox Picture Sequence Memory Test: Unadjusted Scale Score | Episodic Memory | 222 |
| 4 | PicSeq_AgeAdj | NIH Toolbox Picture Sequence Memory Test: Age-Adjusted Scale Score | Episodic Memory | 223 |
| 5 | CardSort_Unadj | NIH Toolbox Dimensional Change Card Sort Test: Unadjusted Scale Score | Executive Function/ Cognitive Flexibility | 224 |
| 6 | CardSort_AgeAdj | NIH Toolbox Dimensional Change Card Sort Test: Age-Adjusted Scale Score | Executive Function/ Cognitive Flexibility | 225 |
| 7 | Flanker_Unadj | NIH Toolbox Flanker Inhibitory Control and Attention Test: Unadjusted Scale Score | Executive Function/ Inhibition | 226 |
| 8 | Flanker_AgeAdj | NIH Toolbox Flanker Inhibitory Control and Attention Test: Age-Adjusted Scale Score | Executive Function/ Inhibition | 227 |
| 9 | PMAT24_A_CR | Penn Progressive Matrices: Number of Correct Responses (PMAT24_A_CR) | Fluid Intelligence | 228 |
| 10 | PMAT24_A_SI | Penn Progressive Matrices: Total Skipped Items (PMAT24_A_SI) | Fluid Intelligence | 229 |
| 11 | PMAT_A_RTCR | Penn Progressive Matrices: Median Reaction Time for Correct Responses (PMAT24_A_RTCR) | Fluid Intelligence | 230 |
| 12 | ReadEng_Unadj | NIH Toolbox Oral Reading Recognition Test: Unadjusted Scale Score | Language/Reading | 231 |
| 13 | ReadEng_AgeAdj | NIH Toolbox Oral Reading Recognition Test: Age-Adjusted Scale Score | Language/Reading | 232 |
| 14 | PicVocab_Unadj | NIH Toolbox Picture Vocabulary Test: Unadjusted Scale Score | Language/Vocabulary | 233 |
| 15 | PicVocab_AgeAdj | NIH Toolbox Picture Vocabulary Test: Age-Adjusted Scale Score | Language/Vocabulary | 234 |
| 16 | ProcSpeed_Unadj | NIH Toolbox Pattern Comparison Processing Speed Test: Unadjusted Scale Score | Processing Speed | 235 |
| 17 | ProcSpeed_AgeAdj | NIH Toolbox Pattern Comparison Processing Speed Test: Age-Adjusted Scale Score | Processing Speed | 236 |
| 18 | VSPLOT_TC | Variable Short Penn Line Orientation: Total Number Correct (VSPLOT_TC) | Spatial Orientation | 251 |
| 19 | VSPLOT_CRTE | Variable Short Penn Line Orientation: Median Reaction Time Divided by Expected Number of Clicks for Correct (VSPLOT_CRTE) | Spatial Orientation | 252 |
| 20 | VSPLOT_OFF | Variable Short Penn Line Orientation: Total Positions Off for All Trials (VSPLOT_OFF) | Spatial Orientation | 253 |
| 21 | SCPT_TP | Short Penn Continuous Performance Test: True Positives = Sum of CPN_TP and CPL_TP (SCPT_TP) | Sustained Attention | 254 |
| 22 | SCPT_TN | Short Penn Continuous Performance Test: True Negatives = Sum of CPN_TN and CPL_TPN (SCPT_TN) | Sustained Attention | 255 |
| 23 | SCPT_FP | Short Penn Continuous Performance Test: False Positives = Sum of CPN_FP and CPL_FP (SCPT_FP) | Sustained Attention | 256 |
| 24 | SCPT_FN | Short Penn Continuous Performance Test: False Negatives = Sum of CPN_FN and CPL_FN (SCPT_FN) | Sustained Attention | 257 |
| 25 | SCPT_TRPRT | Short Penn Continuous Performance Test: Median Response Time for True Positive Responses (SCPT_TPRT) | Sustained Attention | 258 |
| 26 | SCPT_SEN | Short Penn Continuous Performance Test: Sensitivity = SCPT_TP/(SCPT_TP + SCPT_FN) (SCPT_SEN) | Sustained Attention | 259 |
| 27 | SCPT_SPEC | Short Penn Continuous Performance Test: Specificity = SCPT_TN/(SCPT_TN + SCPT_FP) (SCPT_SPEC) | Sustained Attention | 260 |
| 28 | SCPT_LRNR | Short Penn Continuous Performance Test: Longest Run of Non-Responses (SCPT_LRNR) | Sustained Attention | 261 |
| 29 | IWRD_TOT | Penn Word Memory Test: Total Number of Correct Responses (IWRD_TOT) | Verbal Episodic Memory | 262 |
| 30 | IWRD_RTC | Penn Word Memory Test: Median Reaction Time for Correct Responses (IWRD_RTC) | Verbal Episodic Memory | 263 |
| 31 | ListSort_Unadj | NIH Toolbox List Sorting Working Memory Test: Unadjusted Scale Score | Working Memory | 264 |
| 32 | ListSort_AgeAdj | NIH Toolbox List Sorting Working Memory Test: Age-Adjusted Scale Score | Working Memory | 265 |
| 33 | Language_Task_Acc | Language Task OVERALL Accuracy | Language Task | 510 |
| 34 | Relational_Task_Acc | Relational Task OVERALL Accuracy | Relational Task | 518 |
| 35 | WM_Task_Acc | Working Memory Task OVERALL Accuracy | Working Memory Task | 545 |

**Supplementary File 1b** Summary of model performance. The table shows the average performance and the range of the correlation between model-predicted and actual values (r) in deconfounded space, the coefficient of determination (R^2^) in deconfounded space, and of the normalised maximum errors (NMAXAE) in original space.

| **Method** | | **Correlation coefficient (*r*)** | | | | **Coefficient of determination (*R^2^*)** | | | | **NMAXAE** | | | |
| --- | --- | --- | --- | --- | --- | --- | --- | --- | --- | --- | --- | --- | --- |
|  | | **min.** | **mean** | **median** | **max.** | **min.** | **mean** | **median** | **max.** | **min.** | **mean** | **median** | **max.** |
| **Fisher** | Linear | -0.28 | 0.19 | 0.19 | 0.67 | -4.89 | -0.01 | 0.01 | 0.40 | 0.04 | 0.50 | 0.48 | 1.00 |
|  | Gaussian | -0.50 | 0.17 | 0.16 | 0.68 | -9.99E+02 | -0.01 | 0.02 | 0.41 | 0.02 | 0.49 | 0.48 | 18.28 |
| **Naïve** | Linear | -0.41 | 0.05 | 0.05 | 0.52 | -16.04 | -0.05 | 0.00 | 0.26 | 0.02 | 0.55 | 0.50 | 10.27 |
|  | Gaussian | -0.34 | 0.11 | 0.10 | 0.63 | -5.59E+03 | -0.19 | 0.00 | 0.38 | 0.03 | 0.51 | 0.49 | 42.81 |
| **Naïve norm.** | Linear | -0.28 | 0.15 | 0.14 | 0.71 | -2.06 | 0.00 | 0.01 | 0.40 | 0.03 | 0.50 | 0.48 | 1.00 |
|  | Gaussian | -0.36 | 0.09 | 0.09 | 0.62 | -2.37E+08 | -6.85E+03 | 0.00 | 0.39 | 0.02 | 0.96 | 0.49 | 1.22E+04 |
| **KL div.** | | -0.32 | 0.16 | 0.16 | 0.65 | -1.15E+05 | -6.30 | 0.01 | 0.41 | 0.02 | 0.54 | 0.49 | 1.03E+02 |
| **ta KL div.** | | -0.31 | 0.19 | 0.19 | 0.67 | -4.21E+04 | -2.27 | 0.03 | 0.45 | 0.02 | 0.53 | 0.49 | 98.65 |
| **Log-Euclidean** | | -0.48 | 0.14 | 0.13 | 0.61 | -0.24 | 0.03 | 0.01 | 0.31 | 0.02 | 0.50 | 0.48 | 0.99 |
| **Ridge reg.** | | -0.41 | 0.11 | 0.10 | 0.66 | -9.30 | 0.02 | 0.00 | 0.33 | 0.02 | 0.51 | 0.48 | 5.89 |
| **Ridge reg. (Riem.)** | | -0.31 | 0.22 | 0.23 | 0.74 | -0.20 | 0.07 | 0.04 | 0.49 | 0.02 | 0.49 | 0.47 | 0.99 |
| **Selected Edges** | | -0.36 | 0.08 | 0.08 | 0.52 | -2.96 | -0.02 | -0.01 | 0.21 | 0.02 | 0.50 | 0.49 | 0.99 |

­
